# Genistein Antagonizes 17β-Estradiol Effects on Glutamate-Evoked Masseter Muscle Hypernociception in Rats

**DOI:** 10.3389/fneur.2018.00649

**Published:** 2018-08-09

**Authors:** Hui-Fei Jie, Guang-Ju Yang, Rui-Yun Bi, Si-Yi Mo, Ye-Hua Gan, Qiu-Fei Xie

**Affiliations:** ^1^Department of Prosthodontics, Peking University School and Hospital of Stomatology, Beijing, China; ^2^Center for Oral Functional Diagnosis, Treatment and Research, Peking University School and Hospital of Stomatology, Beijing, China; ^3^Third Dental Center, Peking University School and Hospital of Stomatology, Beijing, China; ^4^Central Laboratory and Center for TMD & Orofacial Pain, Peking University School and Hospital of Stomatology, Beijing, China

**Keywords:** genistein, 17β-estradiol, orofacial pain, NMDAR, ERK1/2, hippocampus

## Abstract

Temporomandibular disorders (TMDs) predominantly affect women of reproductive ages, with pain as the main symptom. The aim of the present study was to examine the effects of 17β-estradiol (E2) on glutamate-evoked hypernociception of masseter muscle and to examine whether genistein could antagonize the effects of E2 in female rats. Injection of glutamate into the masseter muscle dose-dependently decreased head withdrawal thresholds, a parameter for mechanical hypernociception. Head withdrawal thresholds in ovariectomized rats also decreased with increasing doses of E2 replacement, and were further aggravated by injection of glutamate (1M, 40μL) into the masseters. Genistein at doses of 7.5 and 15 mg/kg antagonized E2-induced hypernociception of masseter muscle, and at doses of 7.5, 15, and 30 mg/kg also antagonized E2 potentiation of glutamate-evoked hypernociception of masseter muscle. Genistein produced optimal antagonistic effects of E2 on nociception behavior at a dose of 15 mg/kg. On the molecular level, tyrosine phosphorylation of the NR2B subunit of the N-methyl-D-aspartate receptor (pNR2B) and phosphorylated mitogen-activated protein kinase (pERK1/2) were significantly upregulated in the hippocampus following glutamate injection and were further potentiated by E2 replacement. Genistein at dose of 15 mg/kg partially reversed E2-potentiated glutamate-evoked upregulation of pNR2B and pERK1/2 expression in the hippocampus. These results indicated that moderate doses of genistein could antagonize E2 enhanced glutamate-evoked hypernociception of masseter muscle possibly via N-methyl-D-aspartate receptor and ERK1/2 signaling pathways in the hippocampus.

## Introduction

Temporomandibular disorders (TMDs) are characterized by pain in the jaw muscles and/or temporomandibular joint (TMJ). The prevalence of TMDs is higher among women and highest during the reproductive years (1). Although the observed sex differences in TMDs involve multiple factors, estradiol (E2) is likely an important contributor (2). In addition, ~50–70% of patients with TMDs have masseter muscle pain (3). However, whether and how E2 modulates masseter muscle pain of TMDs remains to be fully understood.

Generally, there lacks effective treatments for masseter muscle pain of TMDs. Current pharmacological treatment of muscle pain in TMDs often employs non-steroidal anti-inflammatory drugs (NSAIDs) in combination with a skeletal muscle relaxant. However, there is little evidence of the therapeutic mechanism and there is concern that long term use of NSAID may lead to significant side effects. Therefore, developing novel treatments with more efficient control of masseter muscle pain of TMDs is still clinically necessary. Genistein, a natural phytoestrogen, is structurally similar to E2 and can directly bind to estrogen receptors (ERs) to regulate E2 signaling pathway. It can act as an ER agonist when E2 levels are low or as an ER antagonist when E2 levels are high (4). In the presence of physiological doses of E2, genistein competes with E2 for ER binding (5). Therefore, genistein might be a potential candidate to antagonize the effects of E2 on TMDs related pain.

There are strong evidences that elevated tissue concentrations of glutamate after injury or inflammation may contribute to masseter muscle pain and sensitivity (2). Interstitial glutamate concentrations are 2–4 times greater in the masseter muscles of patients with myofascial TMDs than in healthy control participants (2, 6). Glutamate-evoked pain responses in healthy subjects and patients with TMDs have similar sensory-discriminative and affective-unpleasantness components (7). Furthermore, glutamate-evoked pain of masseter muscles is greater in women than in men, which is consistent with sex differences in the prevalence of TMDs pain (8). Therefore, we raised a question whether genistein could antagonize the effects of E2 on glutamate-evoked masseter muscle pain in the animal model.

The N-methyl-D-aspartate receptor (NMDAR) is one of three subtypes of ionotropic glutamate receptors, and is especially critical to pain processing (9, 10). Pharmacological or genetic inhibition of NMDAR activity effectively alleviates injury-induced pain hypersensitivity both in the central nervous system (CNS) and peripheral nervous system (PNS) (11–14). Furthermore, peripheral inflammation-induced pain hypersensitivity activates the tyrosine phosphorylation of the subunit, NR2B, of NMDAR in the spinal dorsal horn, and intrathecal injection of the selective NR2B antagonist ifenprodil or a Src family tyrosine kinase inhibitor significantly attenuates pain hypersensitivity (15, 16). Peripheral noxious stimulation can activate mitogen-activated protein kinase (ERK1/2) in the CNS, and blockade of ERK1/2 phosphorylation reduces pain hypersensitivity (17, 18). In addition, administration of glutamate into the tongue or whisker pad of rats enhances the head withdrawal reflex, and activates phosphorylated ERK1/2 expression in the CNS (19). Altogether, these studies suggest that the NR2B and ERK1/2 involve in pain hypersensitivity in CNS.

The hippocampus plays a critical role in the sexual dimorphism of pain syndromes, including TMDs. Female rats exhibit higher choline acetyltransferase activity and higher c-fos protein expression in the hippocampus than male rats after injection of formalin into the hind paw (20, 21). Moreover, the human hippocampus also displays sexual dimorphism in pain processing (22). We also previously observed that E2 potentiates the expressions of pain-related genes such as nerve growth factor (NGF) and transient receptor potential vanilloid 1 (TRPV1) in the hippocampus, and enhances the hypernociception of inflamed TMJs in the female rats (23, 24), and that the hippocampus involves in the facilitation of inflammatory pain memory on the occlusal interference-induced masseter muscle hypernociception (25). All these studies suggest that the hippocampus plays a special role in E2-induced TMDs pain.

In this study, we investigated whether genistein could antagonize the effects of E2 on glutamate-evoked hypernociception of masseter muscle and the underlying molecular mechanisms of these effects.

## Materials and methods

### Animals

The experimental protocol was approved by the Animal Care and Use Committee of Peking University (NO. LA201410 Beijing, China), and conformed to the ethical guidelines of the International Association for the Study of Pain. A total of 261 adult female Sprague-Dawley rats (200–220 g; Vital River Laboratory Animal Technology Co. Ltd., Beijing, China) were used in the study. Animals were bred in a specific pathogen-free (SPF) environment and housed at a controlled temperature (22 ± 1°C) on a 12 h light/dark cycle; food and water were available *ad libitum*. All efforts were made to minimize the number of animals used for experiments and their suffering.

### Surgery

Rats were anesthetized by intraperitoneal (i.p.) injections of 1% sodium pentobarbital (50 mg/kg). Then the dorsal lumbar fur was shaved and scrubbed with betadine and alcohol. Immediately, a dorsal midline skin incision was made from caudal to the posterior of the ribs. The ovarian fat pad was gently grasped using forceps until the ovary was exposed and removed. The muscle and skin layer were individually closed and the wound was treated with betadine and antibiotic spray. A high degree of aseptic procedures were maintained throughout the operation. After surgery, the animals were wrapped in a piece of cotton and kept in a heating operation bench for at least 2 h. Sham-operated animals underwent surgical procedures similar to that of OVX rats except that the ovaries were not removed. The effectiveness of OVX or exogenous administration of E2 was confirmed by vaginal smears and hormonal determination. Following OVX and sham operation, all animals were put on soy free diet to exclude the presence of phytosteroids in diet and were allowed to recover for 7 days before group assignment.

### Measurement of head withdrawal thresholds

The head withdrawal threshold was measured as performed in our previous studies (26). Rats were placed in assessment environments 30 min/day to be familiar with the testing surroundings for consecutive 3 days before measuring the baseline of the head withdrawal threshold. The rat was habituated to stand on its hind paws on a soft pad on the table and lean against the experimenter's hand, which wore a regular leather work glove. This test environment was advantageous, in that the rat was not restrained but remained in position.

Nocifensive behavioral responses in the masseter muscles were measured using head withdrawal threshold which was determined by an electronic von-Frey anesthesiometer (Bioseb, FL, USA). The unit was supplied with a rigid plastic tip, capable of transmitting pressure onto the masticatory muscles. We designed a round cap (diameter 3 mm) fixed to the plastic tip in order to diminish the effective skin stimulation, but still produce muscle pressure elicited by the rigid plastic tip of the von-Frey anesthesiometer. When the filament was applied to the midpoint of the masseter muscle, increasing force was gradually applied with the filament oriented perpendicular to the sagittal plane. The force in grams needed to elicit head withdrawal indicative of nociceptive response was recorded five times for each animal. The average of these five values was used as the withdrawal threshold. The baseline of the head withdrawal threshold was recorded for three consecutive days. The investigator was blinded to the treatment conditions in all behavioral experiments.

### Reagents

Monosodium glutamate (G1626, Sigma) was dissolved in sterile isotonic saline (0.9% saline). 17β-estradiol (E-2758, Sigma) was dissolved in ethanol and diluted to 10% in sterile saline immediately before administration. Genistein (253493, J&K Scientific, Beijing, China) was dissolved in 5% dimethyl sulfoxide and 95% polyethylene glycol 400.

### Experimental design

#### Establishment of the masseter muscle pain model

The masseter muscle pain model was established using a glutamate injection. First, the OVX rats were randomly assigned to 5 groups (*n* = 6 per group) and briefly anesthetized using isoflurane, which induces ultrashort anesthesia that lasts only for approximately 30 s after discontinuation (27). The anesthesia was limited to a maximum of 1 min only for injection of glutamate into the masseter muscle. Consistency of the location of injection and even distribution of glutamate or vehicle was ensured using a self-curing resin-based maxillofacial guide with four holes uniformly punched around the midpoint of the masseter muscle. Forty microliter of glutamate at concentrations of 0.1, 0.5, 1 and 2 M or the same volume of vehicle (isotonic saline) was injected using a 30-gauge needle into the masseter muscle of the animals of the five groups, respectively. Head withdrawal thresholds were measured at 5, 15, 30, 45, 60, and 90 min after injections.

Second, OVX rats were also randomly assigned to 5 groups (*n* = 6 per group) and briefly anesthetized and then glutamate (10, 40, or 100 μL of 1 M), hypertonic saline (40 μL of 1 M), or isotonic saline (40 μL) were injected into the masseter muscle of the animals of the five groups, respectively. Head withdrawal thresholds were measured 5, 15, 30, 45, 60, and 90 min after glutamate injections.

#### Measurements of effects of E2 on glutamate-evoked hypernociception of masseter muscle

The 4 groups of OVX rats were treated with E2, administered by subcutaneous abdominal injection daily in the morning, at doses of 0, 20, 80, or 200 μg per rat, respectively, at a volume of 200 μL for 10 days as described previously (23, 28, 29). The naïve and sham OVX groups received subcutaneous abdominal daily injections of the same amount of vehicle. Head withdrawal thresholds were measured on day 8, 9, and 10 post-E2 or vehicle replacement. On day 11, rats in the sham OVX and OVX groups received glutamate (1 M, 40 μL) injections into the masseter muscles and head withdrawal thresholds were measured 15 min after glutamate injection.

After head withdrawal thresholds were measured, all rats were sacrificed using an overdose of sodium pentobarbital. Blood samples were collected and plasma E2 was quantified by using an enzyme-linked immunosorbent assay kit (KGE014; R&D Systems, Minneapolis, MN, USA).

#### Genistein treatments

OVX rats were randomly divided into 7 groups (*n* = 6 per group): no injections, vehicle, and five doses of genistein (2, 7.5, 15, 30, and 60 mg/kg). Genistein or vehicle was injected subcutaneously once per day for 10 days, and the head withdrawal thresholds were measured from day 7 to day10 post-genistein injection.

To examine whether genistein could antagonize the effects of E2 and glutamate on the masseter muscle hypernociception, OVX rats were randomly assigned to 8 groups (*n* = 6, per group): no injections (group A); E2 vehicle + genistein vehicle (group B), 80 μg E2 + genistein vehicle (group C), and 80 μg E2 + 2, 7.5, 15, 30 or 60 mg/kg genistein (groups D, E, F, G, or H, respectively). Group assignments are shown in Table 1. After glutamate injections, groups B, C, D, E, F, G, and H were then renamed B', C', D', E', F', G', and H' (see Table 1). From day 0, group B and C received genistein vehicle, and group D, E, F, G, and H received their respective doses of genistein daily for 12 days. From day 2, genistein or its vehicle was injected 2 h earlier for the related groups, and then group B received E2 vehicle and group C, D, E, F, G, and H received 80 μg E2 for 10 days. On day 12, animals in all groups except group A received glutamate (40 μL of 1 M) injections into the masseter muscles. Head withdrawal thresholds were measured on day −2, −1, 0, 2, 4, 6, 8, 10, and 12, and on day 12 head withdrawal thresholds were measured 15 min after glutamate injection.

**Table 1:** Experimental groups of genistein treatment.

	**Group A**	**Group B (B')**	**Group C (C')**	**Group D (D')**	**GroupE (E')**	**Group F (F')**	**Group G (G')**	**Group H (H')**
ovx	+	+	+	+	+	+	+	+
Gen (mg/kg/d)	−	Vehicle	Vehicle	2	7.5	15	30	60
E2 (μg/d)	−	Vehicle	80	80	80	80	80	80
Glu (1 M, 40 μL)	−	+	+	+	+	+	+	+

*OVX, ovariectomy; Gen, genistein; E2, 17- estradiol; Glu, glutamate; n = 6*.

### Immunohistofluorescence

Rats were deeply anesthetized using sodium pentobarbital (50 mg/kg; i.p.) and transcardially perfused with 250 mL of body-temperature physiological saline (0.9%), followed by 300 mL ice-cold 4% paraformaldehyde in PBS (pH 7.4). The hippocampus was restricted to the area delimited by the rostrocaudal coordinates −2.8 to −4.3 mm relative to bregma (30). These hippocampus were post-fixed in 4% paraformaldehyde for 4 h, incubated in 30% sucrose solution overnight at 4°C, frozen at −20°C, and sectioned into 15-μm-thick slices using a cryostat. The sections were mounted on poly-L-lysine-coated slides, blocked with 10% goat serum and 0.3% Triton-X 100 in PBS for 1 h at room temperature, and incubated with primary antibodies overnight at 4°C. The next day, after extensive washing with PBS, sections were incubated with fluorescein isothiocyanate-conjugated goat anti-rabbit and tetramethyl rhodamine isothiocyanate-conjugated goat anti-mouse secondary antibodies (1:200; Zhong Shan Golden Bridge, Beijing, China) for 2 h in darkness at room temperature. After extensive washing with PBS, the sections were cover slipped with mounting medium containing DAPI. Stained sections were examined under a fluorescence microscope (BX51; Olympus, Tokyo, Japan).

The primary antibodies were as follows: rabbit monoclonal anti-ERα (1:200, ab32063; Abcam, Cambridge, UK), rabbit polyclonal anti-ERβ (1:500, ab3577; Abcam), rabbit polyclonal anti-GPR30 (1:200, ab39742; Abcam), rabbit polyclonal anti-pNR2B (Tyr1472, 1:200; 4208; Cell Signaling Technology, Danvers, MA), rabbit monoclonal anti-pERK1/2 (Thr202/Tyr204, 1:200; 4370; Cell Signaling Technology) and mouse monoclonal anti-NeuN (1:500, ab104224; Abcam).

Immunopositive cells were counted using Image-Pro Plus v. 6.0 (Media Cybernetics, Rockville, MD, USA). Approximately every sixth section was analyzed. To assess the mean numbers of each of the three types of ERs in CA1 and CA3 regions of the hippocampus, the numbers of ERα or ERβ or GPR30 immunoreactive neurons were counted. Percentages of pNR2B and pERK1/2 positive neurons in CA3 were calculated as the number of double-labeled neurons multiplied by 100 and divided by total number of neurons.

### Western blotting

The whole hippocampus of each rat was dissected and homogenized (Ultra-Turrax T10; IKA Laboratory Technology, Staufen, Germany) in ice-cold RIPA lysis buffer (Huaxingbio Science, Beijing, China) with phosphatase and protease inhibitors (04 693 116 001 and 04 693 116 001; Roche, Germany). The homogenate was centrifuged at 13,000 *g* for 20 min at 4°C. The supernatant was collected and protein concentrations were determined using the bicinchoninic acid assay (Beyotime Biotechnology, Shanghai, China). Protein samples were subjected to 6 and 10% sodium dodecyl sulfate polyacrylamide gel electrophoresis and were transferred to polyvinylidene fluoride membranes (Millipore, Bedford, MA, USA). After blocking with 5% non-fat milk for 1 h, the membranes were incubated with anti-pNR2B antibody (1:1,000; Cell Signaling Technology) or anti-pERK1/2 antibody (1:2,000; Cell Signaling Technology) overnight at 4°C. The next day, after washing extensively with Tris-buffered saline containing 0.1% Tween-20, the membranes were incubated with horseradish peroxidase-conjugated secondary antibody (1:5,000; Zhong Shan Golden Bridge) for 1 h at room temperature, developed in enhanced chemiluminescence solution (Beyotime Biotechnology), and examined using a luminescent image analyzer (Fusion FX, Vilber Lourmat, Collegien, France). The membranes were stripped and reprobed with an anti-β-actin antibody to assess the total amount of protein loaded in each lane (1:1,000; Zhong Shan Golden Bridge). The intensities of immuno-positive bands were quantified using ImageJ 1.38 software (National Institutes of Health, Bethesda, MD, USA).

### Statistical analyses

Statistical analyses were performed using SPSS 20.0 (IBM, Armonk, NY, USA) for Windows. Data are expressed as means ± standard deviations for normally distributed data with equal variability. Time course data for head withdrawal thresholds were analyzed using repeated measures analyses of variance (ANOVA). Subsequent pair-wise comparisons were conducted using covariance analyses at each time point. Differences between groups were examined using one-way ANOVA or two-way ANOVA. All multiple-group comparisons were followed by Bonferroni *post hoc* tests. Differences between two groups were examined using independent samples *t*-tests. Differences between two regions from each side of the hippocampus within the groups were assessed using paired *t*-tests. Non-normally distributed data, or data with unequal variability, were evaluated with Mann-Whitney U tests or Kruskal-Wallis analyses of variance followed by Dunn's multiple comparison tests. These data are represented by medians and interquartile ranges. *P* < 0.05 was considered statistically significant. The median half maximal effective concentration (EC50) value was calculated using Prism7.0 software (GraphPad Software Inc., San Diego, CA, USA).

## Results

### Glutamate evoked masseter muscle hypernociception

**Figure 1:**
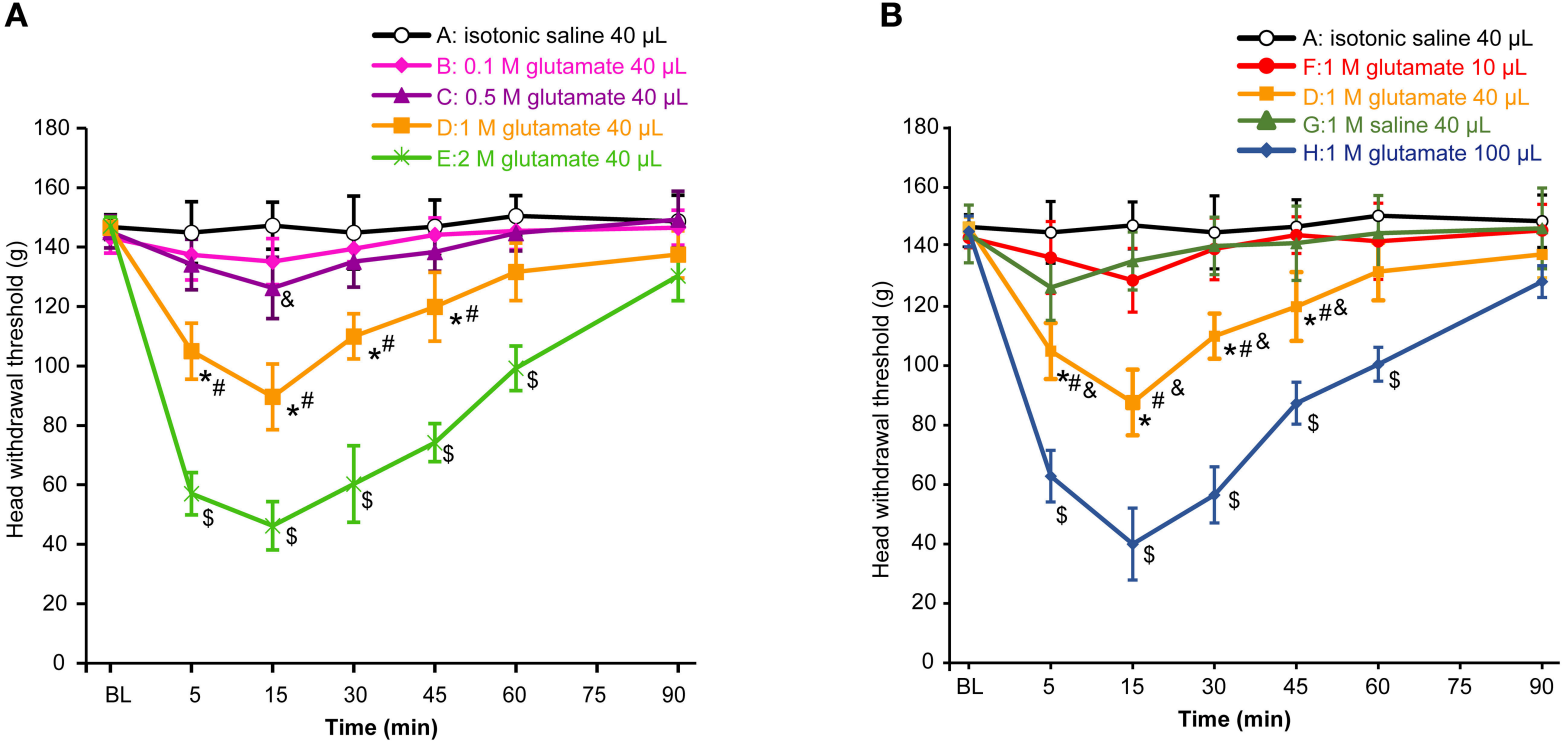
Glutamate evoked hypernociception of the masseter muscle. **(A)** The concentration-dependent effects of glutamate on hypernociception of the masseter muscle. The volume of glutamate injected into masseter muscle was 40 μL in each group. *P < 0.05, comparison between group D and A; ^#^*P* < 0.05, comparison between group D and C; ^$^*P* < 0.05, comparison between group E and D; ^&^*P* < 0.05, comparison between group C and A at 15 min post-injection; **(B)** The volume-dependent effects of glutamate on hypernociception of the masseter muscle. *P < 0.05, comparison between group D and A; ^#^*P* < 0.05, comparison between group D and G; ^&^*P* < 0.05, comparison between group D and F; ^$^*P* < 0.05, comparison between group H and D; *n* = 6, BL, baseline.

First, we assessed whether masseter muscle hypernociception could be generated after injection of different doses of glutamate into the masseter muscle in the OVX rats. As shown in Figure 1A, the head withdrawal threshold was concentration-dependently decreased with injection of glutamate (*P* < 0.05). No significant change in the head withdrawal threshold was noted after isotonic saline injection (group A) as compared with the baseline (*P* > 0.05). The head withdrawal thresholds were not significantly decreased after 0.1 M glutamate injection (group B, *P* > 0.05 vs. group A). Although the nociceptive threshold slightly and transiently decreased at 15 min after 0.5 M glutamate injection (group C, *P* < 0.05 vs. group A), no significant differences in the head withdrawal threshold appeared between group C and A (*P* = 0.085). However, the head withdrawal threshold in group D (1 M glutamate, 40 μL) was significantly decreased at 5 min post-injection (*P* < 0.05), with the lowest level at 15 min post-injection (*P* < 0.01), and recovered virtually to the baseline at 60 min post-injection. A significant lower head withdrawal threshold was observed in group D compared with group C (*P* < 0.05). The nociceptive thresholds in group E (2 M glutamate, 40 μL) was mostly decreased at all-time points examined after injection (*P* < 0.01).

To determine an appropriate glutamate concentration injected into the masseter muscle of the animals, the EC50 value was calculated according to the glutamate concentration effect data. The results showed that the EC50 value was 1.024 M at 15 min post-injection when the head withdrawal thresholds were at the lowest level in all groups. Consequently, 1 M glutamate was used in this pain model and the concentration is consistent with the reported concentration in previous studies for induction of pain both in human and rat masseter muscles (31–33).

As shown in Figure 1B, the head withdrawal threshold in group F (1 M glutamate, 10 μL) was not different from that of group A during 90 min after injection (*P* = 0.159). Instead, 40 μL of 1 M glutamate (group D) significantly decreased the head withdrawal thresholds as mentioned above. To examine whether the osmotic strength might be involved in the effects of glutamate on the hypernociception, 1 M saline (40 μL) was also injected into the masseter muscle (group G) and also showed no different in the head withdrawal threshold from that of group A (*P* = 0.154). Thus, the hypertonicity of the solution did not appear to contribute significantly to the glutamate-evoked mechanical hypernociception. In addition, the head withdrawal thresholds in group H (100 μL of 1 M glutamate) was mostly decreased compared with other groups (*P* < 0.01). Considering that 40 μL of glutamate solution was the minimal volume capable of inducing hypernociception of the masseter muscle, 1 M glutamate 40 μL was used in the subsequent experiments to test the effects of E2 and genistein on hypernociception of masseter muscle.

### E2 induced hypernociception and exacerbated glutamate-evoked masseter muscle hypernociception

**Figure 2:**
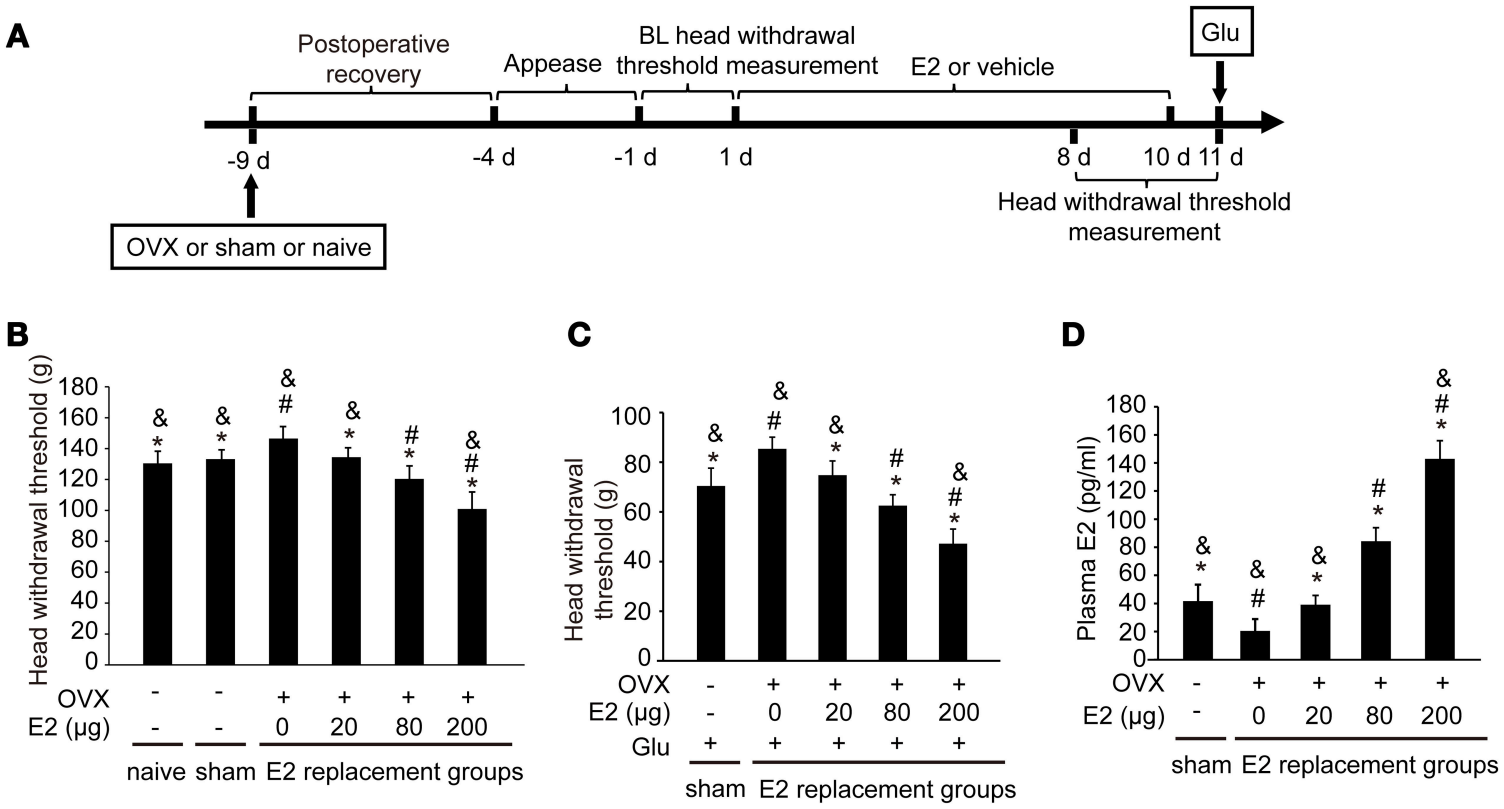
E2 induced mechanical hypernociception and exacerbated glutamate-evoked masseter muscle hypernociception. **(A)** Schematic graph of E2 effects on glutamate-evoked masseter muscle hypernociception. The OVX rats were daily administered with E2 at doses of 0, 20, 80, or 200 μg respectively for 10 days. The naïve and sham OVX groups received daily injections of vehicle. Head withdrawal thresholds were measured on day 8, 9, and 10 post-E2 or vehicle replacement. On day 11, rats in the sham OVX and OVX groups received glutamate injections into the masseter muscles and head withdrawal thresholds were measured 15 min after glutamate injections and then rats were sacrificed and plasma E2 was quantified. **(B)** The effects of E2 on masseter muscle mechanical hypernociception. *P < 0.05 vs. 0 μg E2 group; ^#^*P* < 0.05 vs. 20 μg E2 group; ^&^*P* < 0.05 vs. 80 μg E2 group; **(C)** The effects of E2 on glutamate-evoked masseter muscle mechanical hypernociception. *P < 0.05 vs. 0 μg E2 group; ^#^*P* < 0.05 vs. 20 μg E2 group; ^&^*P* < 0.05 vs. 80 μg E2 group; **(D)** Plasma levels of E2. *P < 0.05 vs. 0 μg E2 group; ^#^*P* < 0.05 vs. 20 μg E2 group; ^&^*P* < 0.05 vs. 80 μg E2 group; *n* = 6. BL, baseline; OVX, ovariectomy; E2, 17β-estradiol; Glu, glutamate.

To examine whether E2 could affect the glutamate-evoked hypernociception of masseter muscle, OVX rats were replaced with various doses of E2 for 10 days before glutamate injection. The experimental design is illustrated in Figure 2A. The baseline of the head withdrawal thresholds for the naïve (130.51 ± 7.83) and sham operated (133.21 ± 5.97) rats were lower than that of the OVX rats (147.23 ± 6.74) (*P* < 0.05, Figure 2B). However, the baseline of the head withdrawal threshold showed no statistical difference between the naïve group and sham group (*P* > 0.05), suggesting that the ovariectomy surgical procedure did not affect the nociceptive behavior response. Hence, to minimize the amount of animals used, the naïve rats were not included in the subsequent experiments. In addition, the head withdrawal thresholds decreased in the OVX rats with increasing doses of E2 replacement (*P* < 0.05). As shown in Figure 2C, E2 dose-dependently exacerbated glutamate-induced decreases in head withdrawal thresholds for the OVX groups (*P* < 0.05). The head withdrawal thresholds in the sham-operated group decreased much lower than the OVX group receiving 0 μg E2 post-glutamate injection (*P* < 0.05).

The plasma E2 increased in OVX rats with increasing doses of E2 replacement (*P* < 0.05, Figure 2D), with the plasma E2 lowest in OVX rats without E2 replacement (*P* < 0.05). Plasma E2 in the sham operated group (41.91 ± 10.34 pg/ml) were higher than that in OVX group with 0 μg E2 (*P* < 0.05), comparable with that in OVX group treated with 20 μg (39.12 ± 6.65 pg/ml; *P* > 0.05), and lower than that in the groups treated with 80 μg and 200 μg (*P* < 0.05). The plasma E2 in the 80 μg group (84.29 ± 9.61 pg/mL) was comparable to that of proestrous rats in our previous study (28), and the plasma E2 in the 200 μg group (142.92 ± 12.9 pg/mL) was similar to that of the rats during the late pregnancy (28). Therefore, 80 μg E2 was used in the subsequent experiments to mimic the plasma E2 level of proestrous rats.

### Dose-dependent differential effects of genistein

#### Genistein affected head withdrawal thresholds in OVX rats without E2 replacement

**Figure 3:**
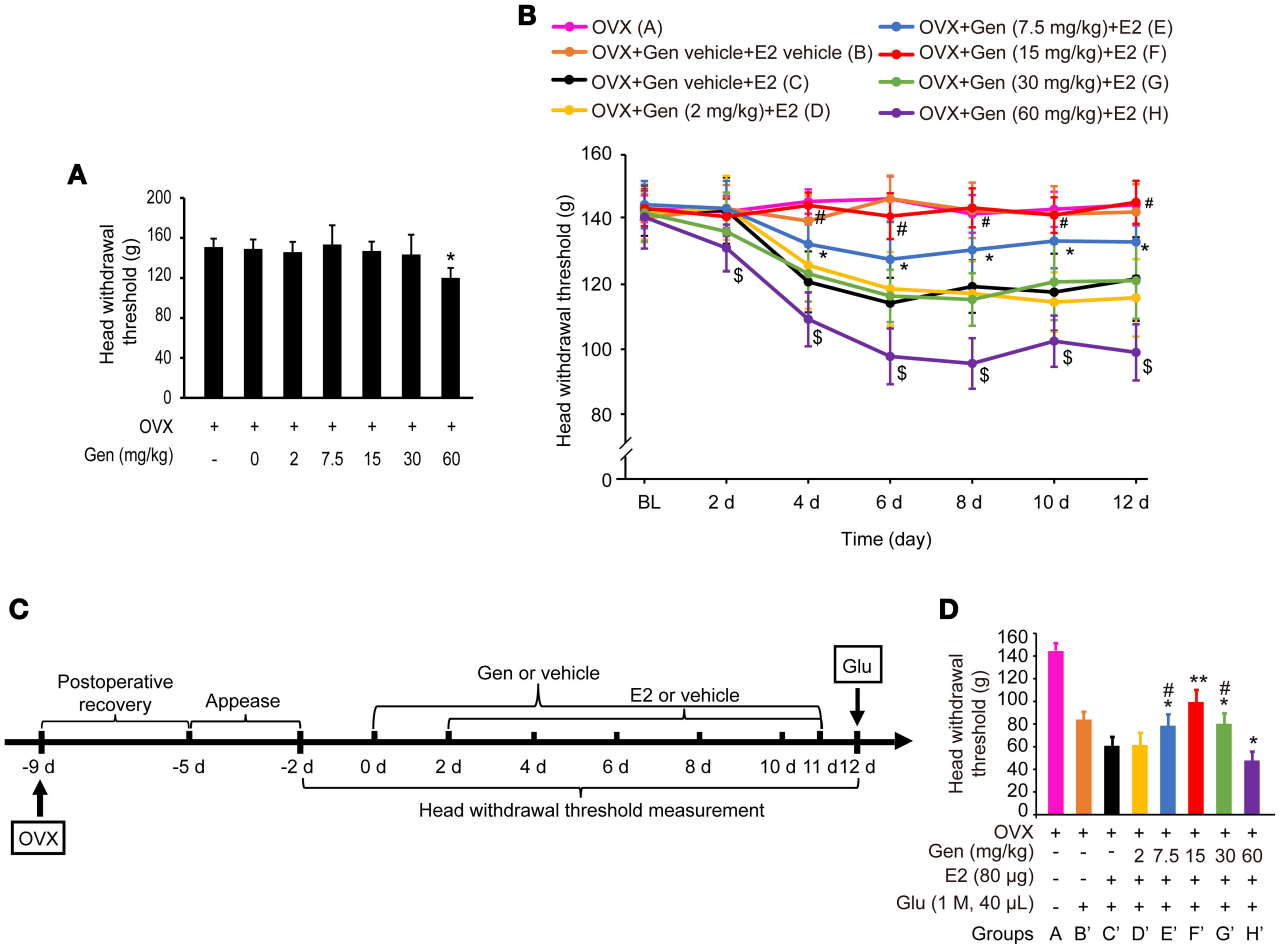
Dose-dependent differential effects of genistein on masseter muscle hypernociception. **(A)** The effects of genistein alone on masseter muscle mechanical hypernociception. *P < 0.05 compared to all other groups. **(B)** The effects of genistein on E2-induced masseter muscle hypernociception. *P < 0.05, comparison between group E and group C; ^#^*P* < 0.05, comparison between group F and group C; ^$^*P* < 0.05, comparison between group H and group C. **(C)** Schematic graph of genistein effects on E2 and glutamate-evoked masseter muscle hypernociception. From day 0, group B and C received genistein vehicle, and group D, E, F, G, and H received their respective doses of genistein daily for 12 days. From day 2, genistein or its vehicle was injected 2 h earlier for the related groups, and then group B received E2 vehicle and group C, D, E, F, G, and H received 80 μg E2 for 10 days. On day 12, animals in all groups except group A received glutamate injections into the masseter muscles. Head withdrawal thresholds were measured on day −2, −1, 0, 2, 4, 6, 8, 10, and 12, and on day 12 head withdrawal thresholds were measured 15 min after glutamate injection. **(D)** The effects of genistein on E2-potentiated glutamate-evoked masseter muscle hypernociception. *P < 0.05, **P < 0.01 vs. group C'; ^#^*P* < 0.05, vs. group F'; *n* = 6. OVX, ovariectomy; Gen, genistein; E2, 17β-estradiol; Glu, glutamate.

As shown in Figure 3A, treatment with genistein at doses of 0, 2, 7.5, 15 and 30 mg/kg did not affect the head withdrawal thresholds compared with the control group (*P* > 0.05), whereas treatment with 60 mg/kg genistein slightly decreased the head withdrawal thresholds compared with the control group (*P* < 0.05).

#### Dose-dependent differential effects of genistein on E2-induced hypernociception of masseter muscle

As shown in Figure 3B, pretreatment with 7.5 mg/kg genistein (group E) partially reversed E2 replacement-induced decrease in the head withdrawal threshold (*P* < 0.05 vs. group C and A), whereas pretreatment with 15 mg/kg genistein (group F) completely reversed E2 replacement-induced decrease in the head withdrawal threshold (*P* < 0.05 vs. group C, and *P* > 0.05 vs. group A). However, pretreatment with genistein at 2 mg/kg (group D) or 30 mg/kg (group G) did not affect E2 replacement-induced decreases in the head withdrawal threshold (*P* = 0.946 vs. group C or *P* = 0.831 vs. group C, respectively). Moreover, pretreatment with 60 mg/kg genistein enhanced E2-induced decreases in head withdrawal thresholds (group H, *P* < 0.05).

#### Dose-dependent differential effects of genistein on E2-potentiated glutamate-evoked hypernociception of masseter muscle

To examine whether genistein could antagonize E2-potentiated glutamate-evoked hypernociception of masseter muscle, various doses of genistein was pretreated before E2 replacement and glutamate injection. The experimental procedure is illustrated in Figure 3C. Pretreatment with 7.5, 15, and 30 mg/kg genistein (Figure 3D, groups E', F', and G', respectively) partially reversed E2-potentiated glutamate-evoked hypernociception (*P* < 0.05 vs. group C'), with the best effect at 15 mg/kg (group F', *P* < 0.05 vs. group E' and G'). However, pretreatment with 60 mg/kg genistein (group H') aggravated E2-potentiated glutamate-evoked hypernociception (*P* < 0.05 vs. group C'). Therefore, 15 mg/kg genistein was selected to explore the moleculare mechanism underlying genistein antagonizing E2-potentiated glutamate-evoked hypernociception of masseter muscle.

### ERβ immunoreactive neurons more abundant than Erα and GPR30 immunoreactive neurons in hippocampus

**Figure 4:**
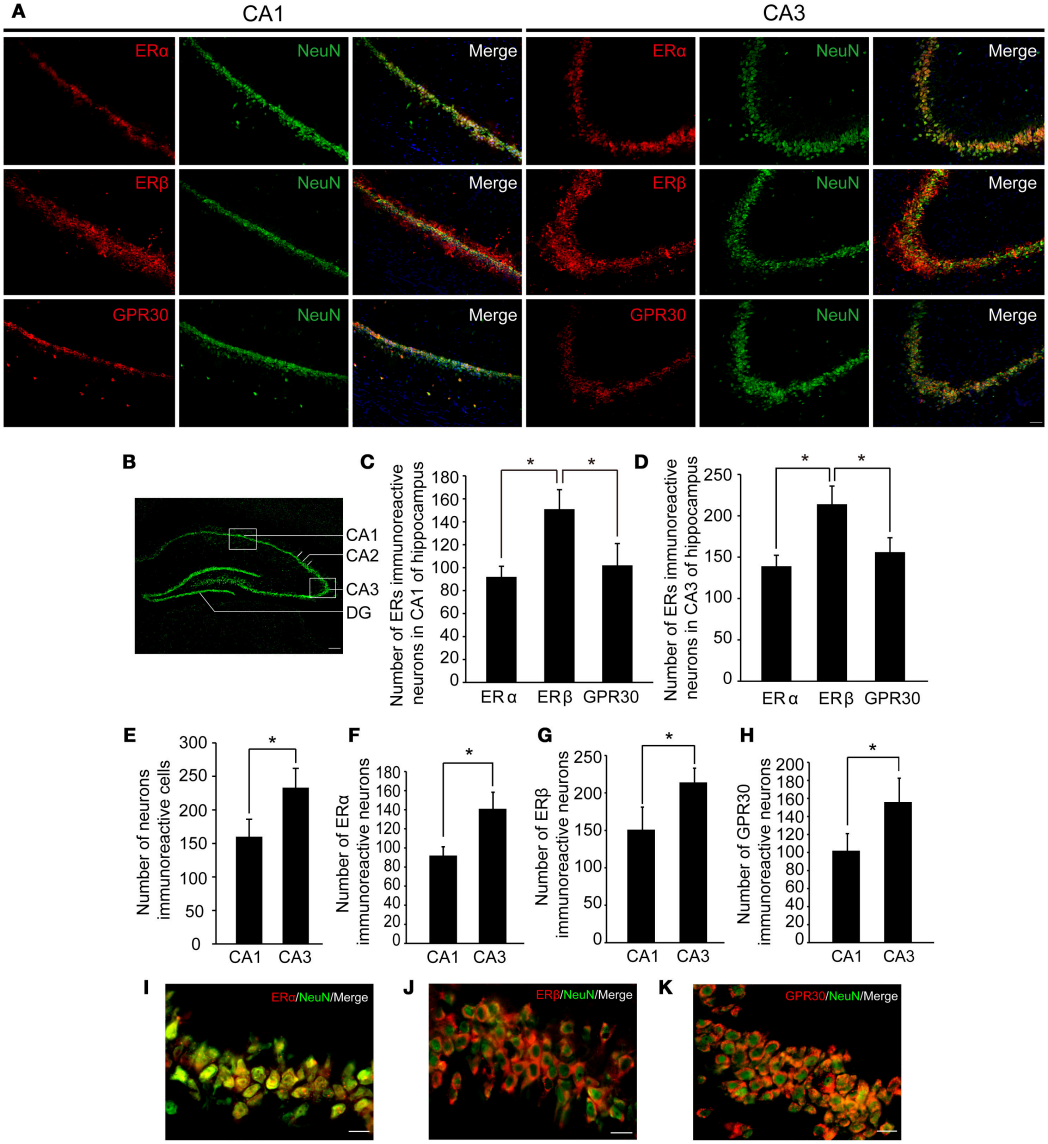
Expression of three types of ERs in hippocampus. **(A)** Immunofluorescence staining in hippocampal CA1 and CA3 regions (boxed region in **B**) showing ERs (red), neurons (green), and nuclei stained with DAPI (blue). Scale bar = 50 μm. **(B)** A portion of one side of the hippocampus marked by NeuN. Scale bar = 200 μm. **(C–H)** The number of ER immunoreactive neurons and neurons in the hippocampal CA1 and CA3 regions; n = 4; *P < 0.05. **(I–K)** Colocalization of ERs with NeuN at higher-magnification views. Scale bar = 20 μm. ER, estrogen receptor; GPR30, G protein-coupled estrogen receptor.

To examine whether the hippocampus was involved in the effects of E2 on glutamate-evoked hypernociception, we first immunostained the estrogen receptors in the hippocampus in OVX rats received 80 μg E2 replacement for 10 days. As shown in Figure 4A, ERα, ERβ, and GPR30 were all expressed in the hippocampus, whereas neuronal nucleus marker (NeuN) was also immunostained to indicate one side of the hippocampus (Figure 4B). As shown in Figures 4C,D, the numbers of ERβ immunoreactive neurons were greater than that of ERα and GPR30 immunoreactive neurons (*P* < 0.05) in both the CA1 and CA3 regions, whereas the latter two were not statistically different between themselves (*P* > 0.05). The numbers of the three ERs immunoreactive neurons and the total neurons were greater in the CA3 region than that in the CA1 region (*P* < 0.05, Figures 4E,F,G,H). Notably, ERα was localized in the nucleus of the neurons (Figure 4I), whereas ERβ and GPR30 were extranuclearly localized in neurons (Figures 4J,K).

### Glutamate injection upregulated pNR2B and pERk1/2 expression in the hippocampus

**Figure 5:**
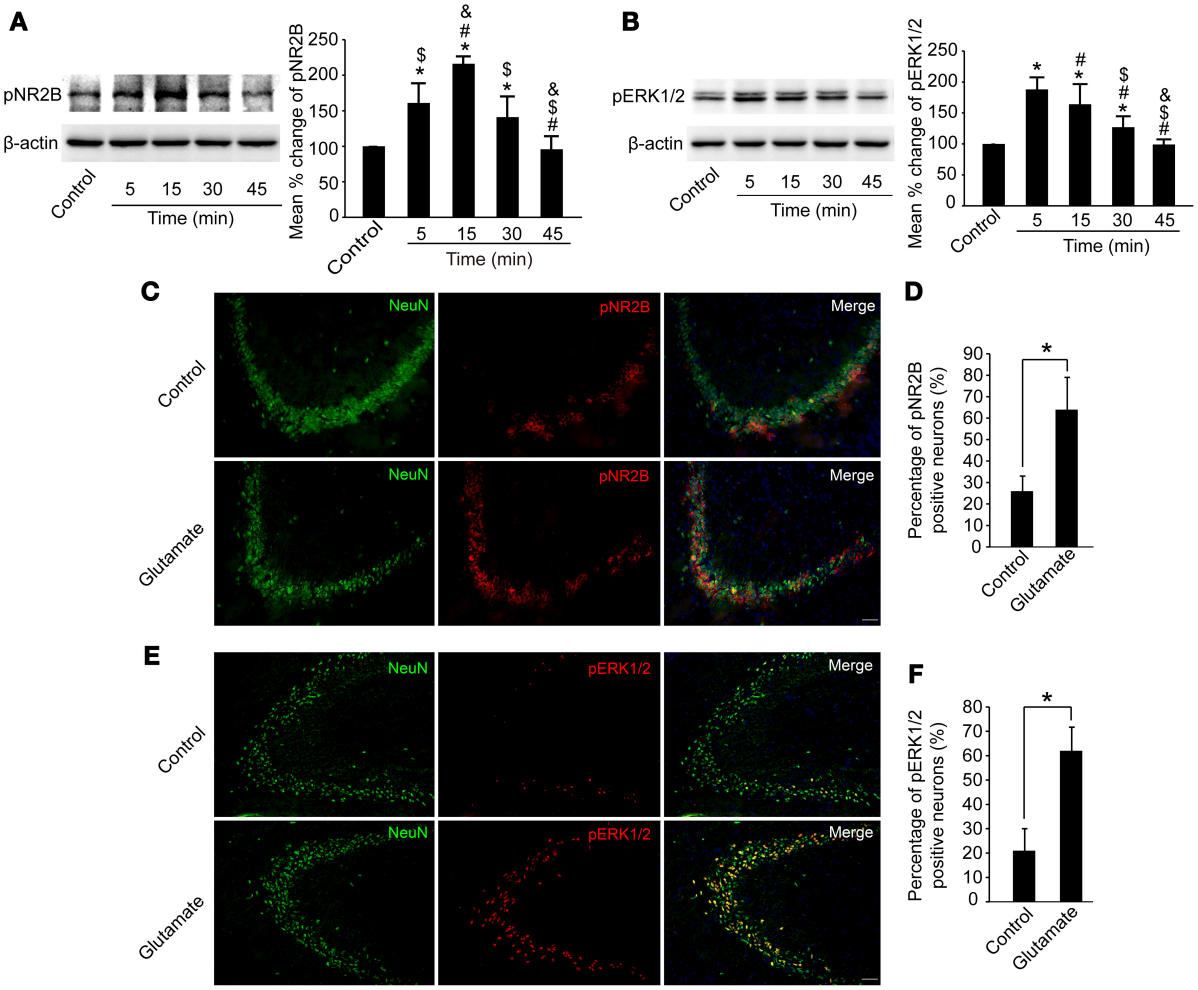
Glutamate injections upregulated pNR2B and pERK1/2 expression in the hippocampus. OVX rats were assigned to 5 groups for western blot analyses. At 5, 15, 30, and 45 min following the glutamate injections, rats were sacrificed and the intact hippocampus was dissected. In addition, in control group, we eliminated the possibility that the needle insertion produced protein expression changes by sacrificing rats immediately after vehicle injections. **(A,B)** Representative western blot of pNR2B and pERK1/2 after glutamate injections. Quantification of protein levels was normalized against loading control β-actin and presented as the relative density compared with the control group. *P < 0.05 vs. control group; ^#^*P* < 0.05 vs. 5 min group; ^$^*P* < 0.05 vs. 15 min group; ^&^*P* < 0.05 vs. 30 min group; *n* = 5, per group. OVX rats were assigned to three groups for immunohistofluorescence staining: (1) rats received isotonic saline injections and were sacrificed immediately for pNR2B and pERK1/2 staining, (2, 3) rats received glutamate injections and were sacrificed respectively after 15 min for pNR2B and 5 min for pERK1/2 staining. **(C,E)** Representative fluorescence photomicrographs showing pNR2B and pERK1/2 positive neurons in CA3 of the hippocampus after glutamate injections. Scale bar = 50 μm. **(D, F)** The percentages of pNR2B and pERK1/2 positive neurons post-glutamate injections; *P < 0.05; *n* = 4, per group. pNR2B, phosphorylated NR2B subunit of the N-methyl-D-aspartate receptor; pERK1/2, phosphorylated (p44/42) mitogen-activated protein kinase.

We then examined whether pNR2B and pERK1/2 in the hippocampus were affected after glutamate injection. As shown in Figures 5A,B, pNR2B were upregulated at 5, 15, and 30 min post-glutamate injections compared to controls (*P* < 0.05), and peaked at 15 min post-injection (*P* < 0.01), whereas pERK1/2 were also upregulated at 5, 15, and 30 min post-glutamate injections compared with controls (*P* < 0.05), and peaked at 5 min post-injection (*P* < 0.01).

We also immunostained pNR2B, pERK1/2, and NeuN in the CA3 region of the hippocampus to assess whether the numbers of pNR2B and pERK1/2 positive neurons were increased after glutamate injection. As shown in Figures 5C–F, the percentage of NeuN-positive neurons expressing pNR2B significantly increased at 15 min post-glutamate injections (*P* < 0.05), whereas the percentage of NeuN-positive neurons expressing pERK1/2 significantly increased at 5 min post-glutamate injections (*P* < 0.05).

### E2 potentiated glutamate-evoked pNR2B and pERK1/2 upregulation in the hippocampus

**Figure 6:**
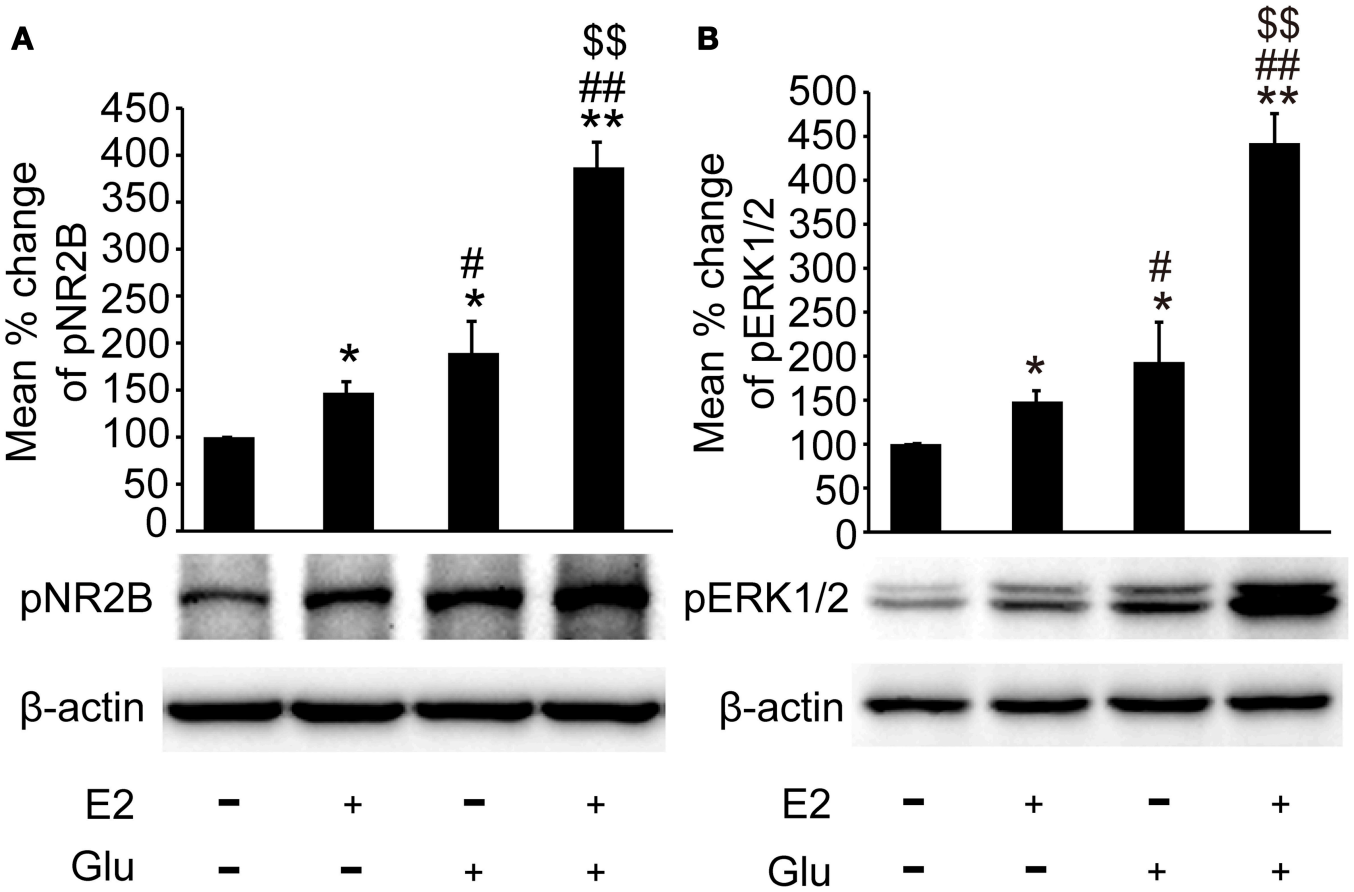
E2 potentiated glutamate-evoked pNR2B and pERK1/2 upregulation in the hippocampus. OVX rats were assigned to 4 groups for western blot analyses: (1) no injections, (2) E2 replacement, (3) glutamate injections, and (4) E2 replacement combined with glutamate injections. **(A, B)** Representative western blots of pNR2B and pERK1/2 after E2 and glutamate treatment. Quantification of protein levels was normalized against loading control β-actin and presented as the relative density compared with the control group. *P < 0.05, **P < 0.01 vs. control group (OVX); ^#^*P* < 0.05, ^##^*P* < 0.01 vs. E2 group; ^$$^*P* < 0.01 vs. Glu group; *n* = 5, per group. OVX, ovariectomy; E2, 17β-estradiol; Glu, glutamate; pNR2B, phosphorylated NR2B subunit of the N-methyl-D-aspartate receptor; pERK1/2, phosphorylated (p44/42) mitogen-activated protein kinase.

As shown in Figures 6A,B, both pNR2B and pERK1/2 expression were significantly upregulated in E2 (*P* < 0.05, vs. controls), and E2 potentiated glutamate induced upregulation of pNR2B and pERK1/2 in the hippocampus (*P* < 0.01 vs. all other groups).

### Genistein pretreatment completely blocked effects of E2 on pNR2B and pERK1/2 in the hippocampus

**Figure 7:**
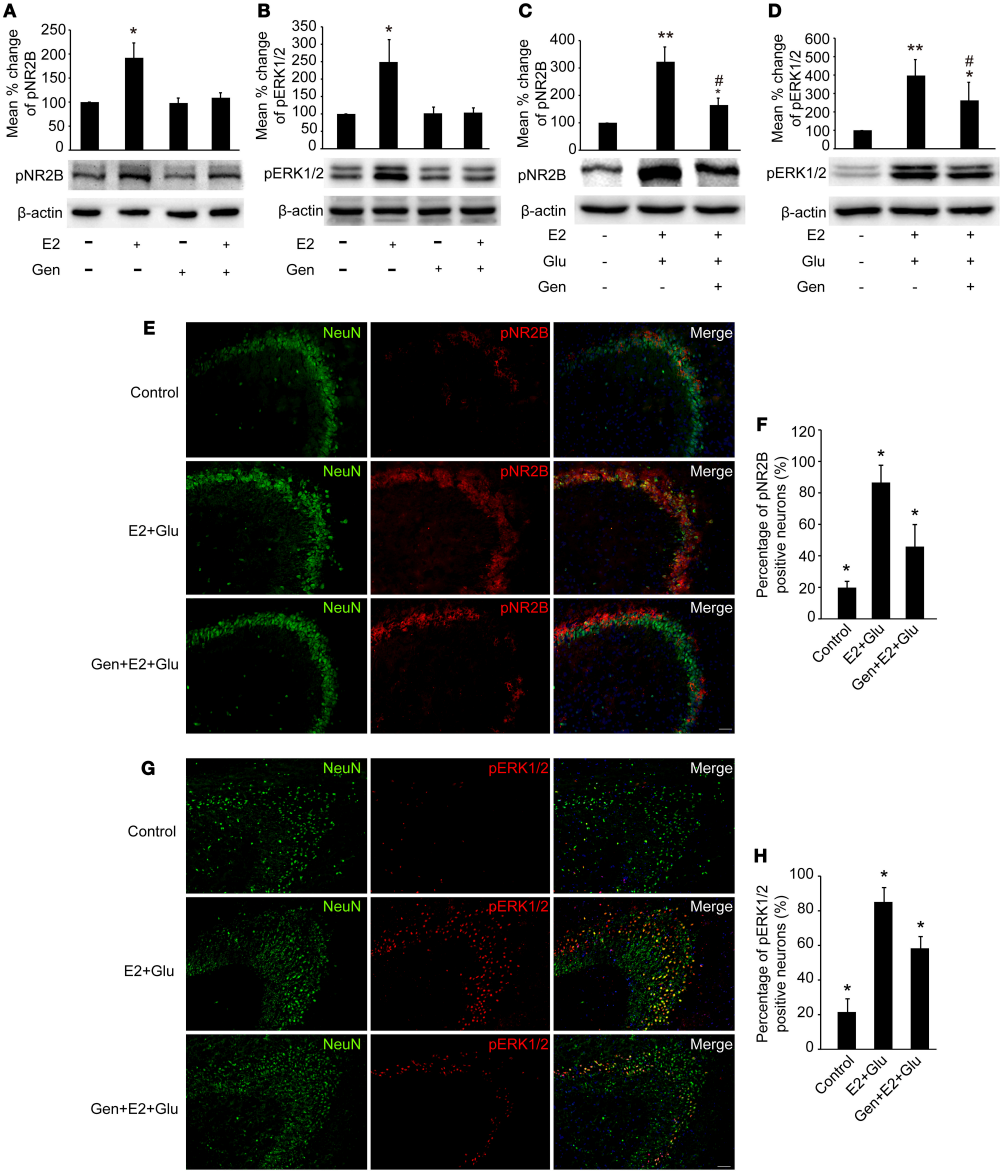
Genistein blocked E2-induced pNR2B and pERK1/2 upregulation, and partially blocked E2-potentiated glutamate-evoked pNR2B and pERK1/2 upregulation in the hippocampus. OVX rats were assigned to 4 groups for western blot analyses: (1) no injections, (2) E2 replacement, (3) genistein treatment, and (4) genistein pretreatment combined with E2 replacement. **(A,B)** Representative western blots of pNR2B and pERK1/2 after E2 and genistein treatment. Quantification of protein levels was normalized against loading control β-actin and presented as the relative density compared with the control group (OVX). *P < 0.05 vs. the rest groups; *n* = 5, per group. To examine the effect of genistein on E2 and glutamate-evoked upregulation of pNR2B and pERK1/2, OVX rats were assigned to 3 groups respectively for protein quantitative detection and immunohistofluorescence staining: (1) no injections, (2) E2 replacement combined with glutamate injections, and (3) genistein pretreatment combined with E2 and glutamate injections. **(C, D)** Representative western blots of pNR2B and pERK1/2 after E2, glutamate and genistein treatment. Quantification of protein levels was normalized against loading control β-actin and presented as the relative density compared with the control group. *P < 0.05, **P < 0.01 vs. control group (OVX); ^#^*P* < 0.05 vs. E2 + Glu group; *n* = 5, per group. **(E,G)** Representative fluorescence photomicrographs showing the pNR2B and pERK1/2 positive neurons in the CA3 region of hippocampus after E2, glutamate and genistein treatment; Scale bar = 50 μm. **(F,H)** The percentages of pNR2B and pERK1/2 positive neurons in the CA3 region of hippocampus after E2, glutamate and genistein treatment; *P < 0.05 comparison among the three groups; n = 4, per group. OVX = ovariectomy; E2 = 17β-estradiol; Glu = glutamate; Gen = genistein; pNR2B = phosphorylated NR2B subunit of the N-methyl-D-aspartate receptor; pERK1/2, phosphorylated (p44/42) mitogen-activated protein kinase; IR, immunoreactive.

As shown in Figures 7A,B, pretreatment with 15 mg/kg genistein completely blocked E2-induced upregulation of pNR2B and pERK1/2 expression (*P* < 0.05), whereas pretreatment with 15 mg/kg genistein alone resulted in no changes in pNR2B and pERK1/2 compared to the controls (*P* > 0.05) in the hippocampus.

### Genistein partially blocked E2-potentiated glutamate-evoked pNR2B and pERK1/2 upregulation in the hippocampus

Densitometry analyses of immunoblots revealed that pretreatment with 15 mg/kg genistein partially blocked E2-potentiated glutamate-induced upregulation of both pNR2B and pERK1/2 expression in the hippocampus (*P* < 0.05, Figures 7C,D).

As shown in Figures 7E–H, immunohistofluorescence also confirmed that E2 potentiated glutamate-induced increase in the percentages of both pNR2B and pERK1/2 immunopositive neurons (*P* < 0.01), which were partially blocked by pretreatment with 15 mg/kg genistein (*P* < 0.05).

## Discussion

In the present study, we demonstrated that genistein at doses of 7.5, 15, and 30 mg/kg effectively antagonized E2-potentiation of glutamate-evoked hypernociception of masseter muscle, and that pNR2B and pERK1/2 in the hippocampus might be involved in genistein antagonizing E2-potentiated glutamate-evoked hypernociception of masseter muscle.

### E2 potentiated glutamate-evoked masseter muscle hypernociception

The plasma levels of E2 were dose-dependently increased with the increasing doses of E2 replacement in the OVX rats; correspondingly E2-induced hypernociception of masseter muscles or the glutamate-evoked hypernociception was also dose-dependently increased with the increasing doses of E2 replacement in the OVX rats. These results suggested that female rats with a higher level of plasma E2 would be more sensitive to mechanical stimuli, especially under the conditions of hypernociception induced by other harmful agents such as glutamate. Blocking ERβ and GPR30 in rats with E2 replacement partially reversed the effects of E2 on glutamate-evoked hypernociception of masseter muscle, suggesting that ERβ and GPR30 were involved in the facilitation of E2 on masseter muscle hypernociception (data not shown). In addition, we also observed that ERβ and GPR30 were primarily distributed at the membrane or in the cytoplasm of hippocampal neurons. There is evidence that E2 binding to plasma and intracellular membrane receptors activates second messenger signaling pathways to modulate receptors and ion channels affecting neuronal activity, which activates transcription factors in seconds to minutes (34). Therefore, it was possible that E2 might amplify glutamate-evoked hypernociception of masseter muscle via ERβ and GPR30 signaling pathway in the hippocampus.

E2 also increased and further aggravated glutamate-evoked pNR2B and pERK1/2 upregulation in the hippocampus, suggesting that the pro-nociceptive effects of E2 might be through NMDAR and ERK1/2 signaling pathway in the hippocampus. Our results were consistent with previous studies, in which E2 aggravates orofacial nociception via NMDAR and ERK1/2 activation in the trigeminal ganglion (35, 36). Moreover, our results also supported the clinical research, which shows that hormone replacement therapy in postmenopausal women increases the risk of developing or exacerbating TMD pain symptoms (37).

### Genistein antagonized E2 enhancement of glutamate-evoked masseter muscle hypernociception

Pretreatment with 7.5, 15, and 30 mg/kg genistein reversed E2 enhanced glutamate-evoked hypernociception of masseter muscle, suggesting that genistein would be potential candidate to clinically antagonize the effects of E2 in TMD related pain. Genistein is a kind of weak plant estrogens that can act as an ER agonist when E2 levels are low and as an ER antagonist when E2 levels are high. However, the binding affinity of phytoestrogens to ERs is 100-1000 fold weaker than E2 (4). Therefore, phytoestrogens compete with ER binding sites leading to reducing the biological effects of E2. The transactivation by premenopausal doses of E2 is inhibited by the isoflavonoids, and the inhibitory effects on E2 activity are more prominent for transfection with ERβ compared to ERα (4). Furthermore, our immunohistofluorescence data also demonstrated that the numbers of ERβ immunoreactive neurons were higher compared to both ERα and GPR30 in the hippocampus. Coincidentally, genistein specifically binds to ERβ, with 7-8 times greater affinity for ERβ than for ERα (38). Therefore, it is possible that genistein has a neuroprotective role as an ERβ antagonist in the hippocampus, thereby alleviating E2-potentiated glutamate-evoked hypernociception of masseter muscle.

However, our data also demonstrated that 60 mg/kg genistein could even induce mechanical hypernociception in the masseter muscles and further enhanced the effects of E2 on masseter muscle nociception. Considering that genistein has both weak estrogenic and anti-estrogenic effects, it is noteworthy that high dose of genistein would have E2-like effects. The dose dependent differential effects of genistein in the present study suggested the clinical application of genistein to antagonize E2 effects would be complicated.

Overall, the effects of genistein on E2-potentiated glutamate-evoked hypernociception of masseter muscle may be related to different E2 contexts, variability in ER subtypes in different tissues, the concentration of genistein in plasma, modalities of genistein treatment (such as diet containing genistein, intragastric administration, subcutaneous injection etc.), dosing frequencies or durations, and different pain models.

### The pNR2B and pERK1/2 in the hippocampus might be involved in genistein antagonizing E2-potentiated glutamate-evoked hypernociception of masseter muscle

In contrast to TMJ pain, there is often a poor correlation between the severity of masseter muscle pain and evidence for definitive tissue pathology. This has led to the concept that TMD-related masseter muscle pain may largely result from alterations in the CNS. The hippocampus is generally not recognized as a major brain area involved in pain processing, however, increasing evidences suggests that the hippocampus contributes to pain awareness. Intra-hippocampal injections of local anesthetic decrease nociceptive behaviors in animals (39), and hippocampal lesions produce avoidance task impairments in rats (25, 40). In support of these suggestions, we also provided additional data here showing that glutamate-evoked hypernociception of masseter muscle result in a significant increase of pNR2B and pERK1/2 expression in the hippocampus which was also potentiated by E2.

Furthermore, genistein partially reversed E2-potentiated glutamate-evoked pNR2B and pERK1/2 upregulation in the hippocampus, and behaviorally antagonized E2 potentiation on glutamate-evoked masseter muscle mechanical hypernociception. Our results are consistent with a recent study, in which genistein attenuates chronic visceral pain by inhibiting pNR2B in the hippocampus of rats (41). In addition, intrathecal pretreatment with genistein abolishes inflammation-induced increases in pNR2B and delays hyperalgesia and allodynia in rats (15). Moreover, genistein directly inhibits NMDA receptors in whole-cell NMDA-activated currents recorded from mouse hippocampal slice culture (42).

Considering that E2 facilitates pain hypersensitivity via ERK1/2 activation in both the PNS and CNS and that genistein is a modulator of ERs, we suggest that the antagonistic effects of genistein also occur via competitive antagonism at ERs, thereby blocking the effects of E2 on ERK1/2 phosphorylation. Although, there is no direct evidence that genistein attenuates pain hypersensitivity by inhibiting ERK1/2 phosphorylation, studies of gastric cancer cells demonstrate that genistein enhances chemosensitivity by suppressing ERK 1/2 activity (43). Furthermore, there is evidence that genistein has anti-diabetic functions via suppressing ERK1/2 pathway in pancreatic β-cells (44). Thus, in the current study, we provide novel possible molecular mechanisms that the antagonistic effects of genistein likely occur via reversing ER induced potentiation of pNR2B and pERK1/2 expression in the hippocampus.

## Conclusions

Our results showed that E2 enhanced glutamate-evoked hypernociception of masseter muscle, which was antagonized by moderate doses of genistein, and that genistein reversed the potentiation of E2 on glutamate-evoked upregulation of pNR2B and pERK1/2 expression in the hippocampus. Genistein could be a potential candidate in the treatment of craniofacial muscle pain.

## Ethics statement

This study was carried out in accordance with the recommendations of International Association for the Study of Pain, Animal Care, and Use Committee of Peking University. The protocol was approved by the Animal Care and Use Committee of Peking University.

## Author contributions

H-FJ, G-JY, S-YM, and R-YB conducted the animal and laboratory experiments. H-FJ and G-JY contributed to the data analyses. H-FJ wrote a draft of the manuscript. Q-FX and Y-HG contributed to the design of the study, the coordination of all experiments, and to critical review of the manuscript.

### Conflict of interest statement

The authors declare that the research was conducted in the absence of any commercial or financial relationships that could be construed as a potential conflict of interest. The reviewer AC and handling Editor declared their shared affiliation.

## Abbreviations

TMDs: temporomandibular disorders
TMJ: temporomandibular joint
E2: 17β-estradiol
ER: estrogen receptor
ERα: estrogen receptor alpha
ERβ: estrogen receptor beta
GPR30: G protein-coupled estrogen receptor
OVX: ovariectomized/ovariectomy
NMDAR: N-methyl-D-aspartate receptor
pNR2B: tyrosine phosphorylation (Tyr-1472) of the NR2B subunit of the NMDAR
pERK1/2: phosphorylated (p44/42) mitogen-activated protein kinase
CNS: central nervous system
PNS: peripheral nervous system.
